# Use of vancomycin as a surrogate for dalbavancin *in vitro* susceptibility testing: results from the DISCOVER studies

**DOI:** 10.1186/s12941-015-0081-5

**Published:** 2015-04-02

**Authors:** Michael W Dunne, Dan Sahm, Sailaja Puttagunta

**Affiliations:** Durata Therapeutics, Inc., Branford, CT, Branford, CT USA; International Health Care Associates, Inc, Washington, DC USA

**Keywords:** Susceptibility testing, Vancomycin, Dalbavancin, Antimicrobial agents, Acute bacterial skin and skin structure infections

## Abstract

**Background:**

Dalbavancin is a lipoglycopepetide antibiotic with activity against gram positive pathogens recently approved for treatment of acute bacterial skin and skin structure infections. Pending the introduction of antimicrobial susceptibility tests, we examined the utility of vancomycin inhibitory concentrations to predict dalbavancin susceptibility in a panel of isolates obtained from phase 3 registration studies.

**Findings:**

99.6% of *Staphylococcus aureus* and 99.0% of beta-hemolytic streptococci which are susceptible to vancomycin will have an MIC at or below the US FDA susceptibility breakpoint for dalbavancin.

**Conclusion:**

Vancomycin should be considered as a surrogate for *in vitro* dalbavancin susceptibility testing.

With the introduction of new antimicrobial agents comes a need for diagnostic tests available in the community which can confirm the *in vitro* susceptibility of target pathogens. Those diagnostic tests, however, require a significant amount of research and development and can only be approved once the susceptibility breakpoints for the agent are identified. Consequently, many antibacterial agents newly available for clinical use are not included in the automated testing systems commonly used in hospital settings.

Dalbavancin is a new lipoglycopeptide with activity against Gram-positive pathogens that has a long half-life allowing for infrequent dosing. Based on two phase 3 clinical trials [1] dalbavancin was recently approved in the United States for treatment of acute bacterial skin and skin structure infections (ABSSSI) dosed intravenously as 1000 mg followed one week later by 500 mg [2] (USPI). Antimicrobial susceptibility testing methodologies in broth have been established by the Clinical and Laboratory Standards Institute [3] (CLSI) however commercially available diagnostics are not presently available. In this circumstance, the use of a surrogate agent to assess for *in vitro* susceptibility is well precedented and has significant utility pending the addition of the new antibacterial agent to an established diagnostic testing susceptibility platform [3,4].

Dalbavancin shares a similar mechanism of action with vancomycin, though providing a significant enhancement in potency, presumably a result of modification to one of the side chains as well as the addition of a lipid tail. The use of vancomycin as a surrogate for dalbavancin *in vitro* susceptibility has been previously proposed [5], and recently repeated [6] (R.N. Jones, D.J. Farrel, R.K. Flamm, submitted for publication), based on a large collection of isolates obtained from a hospital surveillance program. Recently, *in vitro* susceptibility data, derived from the DISCOVER clinical development program has also become available that allows for cross tabulation of mean inhibitory concentrations for these two agents.

## Findings

Isolates derived from patients enrolled in the dalbavancin clinical development program for ABSSSI were tested for susceptibility against dalbavancin and vancomycin. Identification of isolates was confirmed by MALDI Biotyper (Bruker, Bellerica, MA USA). Briefly, dalbavancin was diluted in DMSO and susceptibility testing was performed by broth microdilution in Cation-adjusted Mueller Hinton Broth supplemented with 0.002% (v/v) polysorbate 80, as described in CLSI M7, CLSI M100 and Eurofins Medinet SOP [7] (1-P-PR-PRO-9002355– Broth Microdilution MIC Testing with Frozen Panels). Quality control and interpretations of results were performed according with CLSI M100 with the reference strains *Staphylococcus aureus* ATCC 29213 and *Streptococcus pneumoniae* ATCC 49619. Vancomycin testing was performed in a similar manner, with differences as outlined in Table 1.

**Table 1:** **CLSI methodology for broth microdilution**

**Testing conditions**	**Dalbavancin**	**Vancomycin**
Medium	Cation-adjusted Mueller Hinton Broth supplemented with 0.002% (v/v) polysorbate 80	Cation-adjusted Mueller Hinton Broth
Inoculum	Direct colony suspension, equivalent to a 0.5 McFarland standard	Direct colony suspension, equivalent to a 0.5 McFarland standard
Incubation	35 +/− 2°C; ambient air; 24 hours	35 +/− 2°C; ambient air; 24 hours
Solvent/diluent	DMSO	Water

Of the 634 *S. aureus* isolates identified, the dalbavancin MIC_90_ was 0.06 μg/ml. Three isolates (0.4%) had an MIC greater than the FDA breakpoint of >0.12 μg/ml. The MIC_90_ of *S. aureus* for vancomycin was 1 μg/ml and no isolate had an MIC greater the breakpoint of 2 μg/ml. Cross tabulation of these susceptibility data confirmed that 99.6% of the isolates that were susceptible to vancomycin were also susceptible to dalbavancin (Table 2).

**Table 2:** **Cross tabulation of** ***S. aureus*** **isolates with mean inhibitory concentrations for vancomycin and dalbavancin**

**Vancomycin MIC (μg/ml)**	**Dalbavancin MIC (μg/ml)**					
	**0.008**	**0.015**	**0.03**	**0.06**	**0.12**	**0.25**
0.25		1				
0.5		3	142	191	5	1
1	1	2	49	230	7	1
2						1

Of the 192 beta-hemolytic streptococci, which include *S. pyogenes* (87), *S. agalactiae* (36), *S. dysgalactiae* (4), *S. anginosus* group (49), Group C streptococci (9) and Group G streptococci (7), the dalbavancin MIC_90_ was 0.06 μg/ml. Two isolates had an MIC >0.12 μg/ml. The two isolates (1%) that would be considered non-susceptible to dalbavancin were both susceptible to vancomycin. Cross tabulation of these susceptibility data confirmed that 99.0% of the isolates that were susceptible to vancomycin were also susceptible to dalbavancin (Table 3).

**Table 3:** **Cross tabulation of Beta-hemolytic streptococcal isolates with mean inhibitory concentrations for vancomycin and dalbavancin**

**Vancomycin MIC (μg/ml)**	**Dalbavancin MIC (μg/ml)**								
	**<0.001**	**0.002**	**0.004**	**0.008**	**0.015**	**0.03**	**0.06**	**0.12**	**0.25**
≤0.015	1	1			1				
0.03		1							
0.06				1					
0.12		1							
0.25		2	19	16	38	14	4	3	2
0.5		7	7	18	20	16	3	4	
1			1	4	3		1		

One patient treated with dalbavancin had an MIC that would be considered non-susceptible (MIC = 0.25 μg/ml). This organism was positive for the Panton-Valentine leukocidin toxin and methicillin resistance and the patient was a clinical success at Day 3 and a clinical cure at Day 14. The other patient with a dalbavancin MIC to *S. aureus* of >0.12 μg/ml (MIC = 0.25 μg/ml) was treated with vancomycin and was a clinical success (Table 4).

**Table 4:** **Two patients with** ***S. aureus*** **isolates with a dalbavancin MIC of 0.25 μg/ml**

**Characteristic**	**Patient 1**	**Patient 2**
Treatment Group	Vancomycin/linezolid	Dalbavancin
Age (years)	51	34
Type of infection	Traumatic Wound Infection	Cellulitis
Wound culture	MSSA	MRSA
Vancomycin MIC	1	0.5
PVL toxin	Negative	Positive
mecA gene	Negative	Positive
Clinical response at 48–72 hours	Responder	Responder
Clinical outcome at Day 14	Success	Success

Staphylococcal and beta-hemolytic streptococcal isolates were collected from two large clinical trials supporting the development of dalbavancin for treatment of ABSSSI. Based on recent epidemiologic surveys, the distribution of MICs in this collection is representative of isolates circulating in hospitals in the United States [8]. 99.6% of *S. aureus* and 99.0% of beta-hemolytic streptococci which are susceptible to vancomycin (MIC ≤ 2 μg/ml) will have an MIC at or below the US FDA susceptibility breakpoint for dalbavancin.

While vancomycin susceptible isolates were also susceptible to dalbavancin, no data are available regarding the potential clinical outcome of patients treated with dalbavancin who have isolates with an MIC to vancomycin > 2 μg/ml. Anecdotally, a small number of these patients have been observed in clinical trials and have been successfully treated. While the FDA breakpoint is 0.12 μg/ml, based to a large degree on the clinical outcome data of a sufficient number of patients infected with isolates at that MIC, a preliminary assessment of the ECOFF calculations [9] for *S. aureus* suggests a breakpoint of 0.25 μg/mL, as this is the highest MIC of organisms lacking phenotypically expressed resistance [10]. A reassessment of the existing breakpoint will be enabled by analysis of more patients treated with dalbavancin who had organisms with an MIC > 0.12 μg/ml.

A strong correlation between vancomycin and dalbavancin *in vitro* susceptibility results was observed for both sets of isolates. While broth microdilution methodologies to guide performance of *in vitro* susceptibility testing have been published, these data support the proposal that vancomycin can be used as a surrogate for susceptibility testing of dalbavancin, pending the introduction of dalbavancin into established diagnostic susceptibility testing platforms.

Over 99.6% of *S. aureus* and 99.0% of beta-hemolytic streptococci which are susceptible to vancomycin (MIC ≤ 2 μg/ml) will have an MIC at or below the US FDA susceptibility breakpoint for dalbavancin. Vancomycin should be considered for use as a surrogate for *in vitro* dalbavancin susceptibility testing.

## Availability of supporting data

The data supporting the results of this study are included within this article.
